# Oxidative Stress Related to Mechanical Heart Valves: A Pilot Cross-Sectional Study

**DOI:** 10.3390/antiox14101264

**Published:** 2025-10-20

**Authors:** Ilaria Maria Palumbo, Arianna Pannunzio, Danilo Menichelli, Vittoria Cammisotto, Valentina Castellani, Simona Bartimoccia, Emanuele Valeriani, Vito Maria Daniele Cormaci, Daniele Pastori, Pasquale Pignatelli

**Affiliations:** 1 Department of General and Specialized Surgery and Anesthesiology, Sapienza University of Rome, 00185 Rome, Italy; 2 Department of Medical and Cardiovascular Sciences, Sapienza University of Rome, 00185 Rome, Italy; 3 IRCCS Neuromed, 86077 Pozzilli, Italy

**Keywords:** oxidative stress, mechanical prosthetic heart valves (MPHVs), NOX2, platelets

## Abstract

Background: Valvular heart disease remains a major global health issue, with mechanical prosthetic heart valves (MPHVs) widely used in surgical valve replacement. However, these devices carry a risk of thrombosis, particularly in the mitral position. Several mechanisms may be involved in this risk, but the role of oxidative stress (OxS) remains unclear. Our aim was to assess the relationship between OxS impairment and platelet activation. Methods: We analyzed data from a pilot, observational, monocentric study conducted at our anticoagulation clinic at Sapienza University of Rome, involving adult patients with MPHVs (aortic or mitral) on vitamin K antagonist therapy, enrolled between June and September 2024. Clinical data and blood samples were collected to evaluate markers of NOX2-mediated OxS (sNOX2-dp, H_2_O_2_) and platelet activation (sCD40L) using ELISA-based assays. Results: Our cohort included 30 patients with mitral MPHVs and 30 patients with aortic MPHVs (46.7% males, 53.3% females). Serum sNOX2-dp and H_2_O_2_ were significantly higher in patients with mitral MPHVs (28.69 [25.08–33.18] vs. 24.27 [17.30–26.41] pg/mL, *p* = 0.001, and 22.94 [15.79–27.33] vs. 16.73 [12.50–20.87] µM, *p* = 0.013, respectively) compared with aortic MPHV patients. sCD40L was significantly elevated in mitral versus aortic MPHVs (5.61 [3.69–6.89] vs. 3.65 [2.14–5.54] ng/mL, *p* = 0.009). Spearman’s correlation analysis showed a significant correlation between sNOX2-dp levels and sCD40L in both groups (mitral MPHVs: r_s_ = 0.521, *p* = 0.003; aortic MPHVs: r_s_ = 0.443, *p* = 0.014). Conclusions: Mitral MPHVs are associated with heightened OxS and platelet activation compared to aortic MPHVs. These findings may help explain the higher thrombotic risk observed with mitral valves and support differential management strategies.

## 1. Introduction

Valvular heart disease is a leading cause of cardiopathy, whose epidemiology varies around the world: degenerative disease predominates in developed countries, while rheumatic heart disease is still prevalent in developing countries [1,2,3,4,5]. Surgical valve replacement is the standard of care for treatment in suitable patients, and can be performed with mechanical prosthetic heart valves (MPHVs) or biological ones. MPHVs are more thrombogenic but more durable: the estimated annual rate of mechanical valve thrombosis ranges from 0.1% to 5.7% [6]. For this reason, guidelines recommend lifelong anticoagulant therapy with vitamin K antagonists (VKAs) [7,8]. However, despite anticoagulant therapy, patients with MPHVs are still exposed to a residual thrombotic risk: the estimated event rate is 0.55 (95% confidence interval-CI—0.31–0.98) per 100 person-years in patients with good anticoagulation control, but can reach 1.31 (95% CI: 0.48–3.57) per 100 person-years in poorly controlled patients [9].

The intensity of anticoagulation therapy can be modulated according to lower or higher INR targets, which depend on valve design (bileaflet versus tilting disk or caged-ball), associated risk factors for thromboembolism, and on valve position: in particular, mitral valves show higher thrombotic risk compared with aortic MPHVs, and carrying a mitral MPHV is an acknowledged risk factor for thromboembolism [10]. The reason for this higher thrombotic risk has not been completely explained: pathogenesis of device-induced clotting includes protein absorption, platelet activation, thrombin and complement activation [11], but it is not clear why mitral valves are more thrombogenic than aortic ones.

Previous studies have explored oxidative stress as a risk factor in cardiovascular disease; in particular, NADPH-oxidase 2 (NOX2), an enzyme involved in innate immunity, has been extensively studied in relation to platelet activation and atherothrombosis [12,13]. Its physiological role is linked to bacterial killing in phagocytes through production of radical oxygen species (ROS), but NOX2 is also involved in platelet activation via superoxide anion formation and conversion to H_2_O_2_ [12,13]: experimental data show that ROS influence platelet recruitment and thrombus growth, and, conversely, that in patients with genetically determined NOX2 deficiency, platelet activation is impaired and carotid atherosclerotic burden is reduced [13].

Oxidative stress has already been studied in valvular heart disease [14]: data about rheumatic heart disease [15], aortic valve calcification [16,17,18,19,20,21], and bioprosthetic heart valve dysfunction [22,23] have shown that oxidation is implied in these settings. In particular, superoxide and hydrogen peroxide seem involved in the calcification of stenotic aortic valves in humans, maybe due to a mechanism based on reduction in expression and activity of antioxidant enzymes and modification of nitric oxide synthases (NOS) activity [17,20].

However, specific data on MPHVs are lacking.

- Aims of this study were: to evaluate if patients with mitral MPHVs, who are exposed to higher risk of thrombosis, show higher oxidative stress (OxS) compared with patients with aortic MPHVs;to evaluate if OxS is correlated with enhanced platelet activation in patients with aortic and mitral MPHVs, respectively.

## 2. Materials and Methods

We performed a pilot, nonprofit, observational, monocentric study promoted by the Department of Clinical Internal, Anaesthesiologic and Cardiovascular Sciences of Sapienza University of Rome. We included 60 consecutive patients with MPHVs, either aortic or mitral, referring to the anticoagulation clinic of Policlinico Umberto I, Rome, from 1 June 2024, to 30 September 2024. Inclusion criteria were implantation of aortic or mitral MPHVs at least three months before recruitment, age ≥ 18 years, and signing of written informed consent. Exclusion criteria were implantation of biologic valves, concomitant antiplatelet therapy, infections, active cancer, and history of autoimmune diseases. All patients were on treatment with VKAs, either warfarin or acenocoumarol, since phenprocumon is not available in Italy. During the enrolment, we performed a complete medical visit during which we collected data on site of MPHV, comorbidities, including chronic obstructive pulmonary disease (COPD), heart failure (HF), atrial fibrillation (AF), myocardial infarction (MI), peripheral artery disease (PAD), previous stroke or transient ischemic attack (TIA), concomitant therapy with angiotensin-converting enzyme inhibitors/angiotensin receptor blockers (ACE-i/ARBs), beta-blockers, lipid-lowering drugs (LLD), proton pump inhibitors (PPI), calcium channel blockers (CCB), digoxin, amiodarone, antiarrhythmic, and diuretics.

All patients signed an informed written consent at study entry. The study was approved by the local ethics committee of Sapienza University (no. 0076/2024) and was conducted according to the 1975 Declaration of Helsinki.

### 2.1. Blood Sample Collection and Analysis

Between 8:00 a.m. and 9:00 a.m., after overnight fasting (12 h) and supine rest for at least 10 min, all the patients underwent blood samples. Blood samples obtained from patients were collected into tubes (Vacutainer Systems, Belliver Industrial Estate, Plymouth, UK) with or without anticoagulant (3.8% sodium citrate) and were centrifuged at 300 g for 10 min at room temperature to obtain supernatant. For serum isolation blood was drawn and allowed to clot at room temperature for 1 h. Serum and plasma samples were immediately stored at −80 °C until analysis.

### 2.2. Serum sNOX2-dp Detection

NOX-2-derived peptide (sNOX2-dp) release is a marker of NADPH oxidase activity and was evaluated in serum by an ELISA method as previously described [24]. Briefly, the assay is based on coating of reference standards and samples into a 96-well plate for at least 12 h at 4 °C. After the incubation, an anti-sNOX2dp-horseradish peroxidase (HRP) monoclonal antibody against the amino acidic sequence (224–268) of the extracellular membrane portion of NOX2 is added. The enzyme activity is detected after the addition of the substrate 3,30,5,50-tetramethylbenzidine (TMB) and measured spectrophotometrically at 450 nm. Values related to sNOX2-dp concentration were expressed as pg/mL and intra- and inter-assay coefficients of variation were 5.2% and 6%, respectively.

### 2.3. Hydrogen Peroxide (H_2_O_2_) Production

Hydrogen peroxide (H_2_O_2_) production was evaluated using a colorimetric assay according to manufacturer instructions (Abcam, Cambridge, UK). The reaction product was measured spectrophotometrically at 450 nm and expressed as µM. The intra- and inter-assay coefficients of variations were both <10%.

### 2.4. Soluble CD40 Ligand Detection

Soluble CD40 Ligand (sCD40L) concentration was measured in plasma by an Immunoassay solid-phase ELISA (DRG International, Springfield Township, OH, USA). Values were expressed as ng/mL and the intra-assay and inter-assay coefficients of variation were 3.2% and 4.3%, respectively.

### 2.5. Statistical Analysis

Categorical variables were reported as counts (percentage), and the Pearson chi-square test was used to compare proportions. Continuous variables were expressed as mean and standard deviation (SD) or median and interquartile range (IQR), depending on their distribution, which was assessed by the Kolmogorov−Smirnov test. Descriptive analysis of baseline characteristics was performed according to valve implantation site. Appropriate nonparametric tests (Mann−Whitney U test and Spearman rank correlation test) were used for non-normal variables. Spearman correlation analysis was used to explore correlations between oxidative stress and platelet activity. We then performed a multivariable stepwise linear regression analysis adjusted for potential confounders.

Only *p* values < 0.05 were considered as statistically significant. All tests were two-tailed. Statistical analysis was performed with IBM SPSS25.0 software (SPSSInc., Chicago, IL, USA) and GraphPad—Prism10 (GraphPad Software, 225 Franklin Street. Fl. 26, Boston, MA, USA).

## 3. Results

Population characteristics are shown in Table 1. Mean age was 57.2 [IQR 48.05–64.42] years, and 53.3% of participants were women. There was a significant difference in AF prevalence between patients with aortic and mitral MPHVs, since more patients with mitral MPHVs were affected (60% vs. 24.1%, *p* = 0.005). There were not significant differences regarding ethnicity and cardiovascular risk factors and comorbidities such as arterial hypertension, diabetes mellitus, history of stroke/TIA, PAD, previous MI, dyslipidaemia, cancer, HF, smoke, and alcohol abuse. Patients with aortic MPHVs had more often a history of COPD (20% vs. 0%, *p* = 0.010), while patients with mitral MPHVs had more often a history of thyroid disease (24.1% vs. 3.3%, *p* = 0.020). Warfarin was statistically more prescribed than acenoucumarol (71.7% vs. 28.3%, *p* = 0.045). More patients with mitral MPHVs were treated with ACE-I/ARBs (64% vs. 33.3%, *p* = 0.032) and with antiarrhythmics (40.0% vs. 8.3%, *p* = 0.010) and amiodarone (28.0% vs. 4.2%, *p* = 0.024). There were not significant differences according to treatment with LLD, beta-blockers, CCB, digoxin, diuretics, or PPI.

### Oxidative Stress and Platelet Activation in Mitral and Aortic MPHV

**Figure 1:**
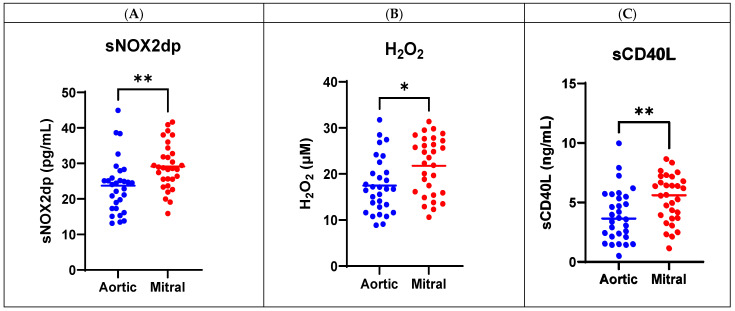
Oxidative stress and platelet activation in aortic and mitral MPHVs. Oxidative stress parameters including (**A**) soluble NOX2-derived peptide (sNOX2-dp), (**B**) hydrogen peroxide (H_2_O_2_), and platelet activation such as (**C**) CD40 ligand (CD40L) in aortic and mitral mechanical prosthetic heart valves (MPHVs). Data were expressed as median ± IQR; *p* values were calculated using Mann–Whitney U test (* *p* < 0.05; ** *p* < 0.01).

Regarding oxidative stress, we observed significantly higher levels of sNOX2dp (28.69 pg/mL [IQR: 25.08–33.18] vs. 24.27 pg/mL [IQR: 17.30–26.41], *p* = 0.001) and H_2_O_2_ (16.73 µM [IQR: 12.50–20.87] vs. 22.94 µM [IQR: 15.79–27.33], *p* = 0.013) in patients with mitral MPHVs compared with aortic MPHVs (Table 2). Platelet activation was enhanced too in patients with mitral MPHVs, according to plasma sCD40L (3.65 ng/mL [IQR: 2.14–5.54] vs. 5.61 ng/mL [IQR: 3.69–6.89], *p* = 0.009) compared to aortic MPHV ones, as shown in Figure 1.

**Figure 2:**
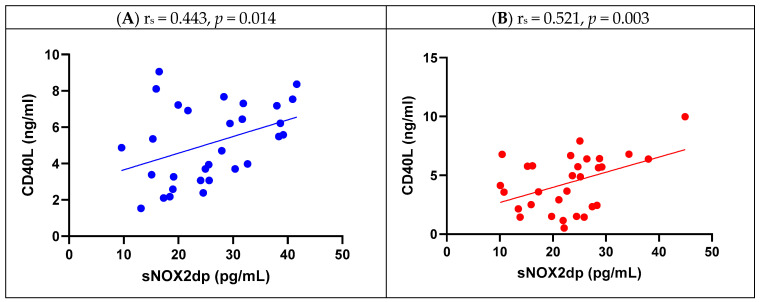
Spearman’s correlation analysis between sNOX2dp and sCD40L in aortic (**A**) and mitral (**B**) MPHVs.

Spearman’s correlation analysis (Table 3) showed a significant correlation between NOX2 activity and H_2_O_2_ levels (r_s_ = 0.720, *p* < 0.001 for aortic, and r_s_ = 0.800, *p* < 0.001 for mitral), and between NOX2 activity and sCD40L (r_s_ = 0.443, *p* = 0.014 for aortic, r_s_ = 0.521, *p* = 0.003 for mitral) as shown in Figure 2.

We then performed a multivariable stepwise linear regression analysis to assess the role of MPHV implantation site on oxidative stress. After adjustment for potential confounders, as reported in Table 4, sNOX2 (B—5.042, *p* = 0.009), H_2_O_2_ (B—3.867, *p* = 0.019), sCD40L (B—1.722, *p* = 0.003) were inversely associated with aortic implantation site, compared to mitral implantation site.

## 4. Discussion

NOX2-mediated oxidative stress was enhanced in our cohort of patients with mitral MPHVs compared with aortic MPHVs, as shown by circulating levels of NOX2 soluble peptide and H_2_O_2_. Platelet activity, measured by sCD40L levels, was also enhanced. A direct correlation between NOX2-mediated oxidative stress and platelet activity is suggested by Spearman analysis.

NOX2 has been extensively studied in patients with cardiovascular risk factors and in patients affected by cardiovascular disease [25,26,27], from vascular dysfunction in peripheral artery disease [28], atrial fibrillation [29] and STEMI [30]. Our hypothesis was that mitral MPHVs could be related to higher risk of thrombosis through platelet activation mediated by oxidative stress.

Baseline characteristics from our cohort showed significant differences between patients with aortic and mitral MPHVs regarding AF, antiarrhythmics, amiodarone, thyroid disease, COPD, and use of warfarin or acenocoumarol and of ACE-I/ARBs. AF is a common complication of cardiac surgery, occurring in 10% to 65% of patients [31]. At the same time, approximately 35% of patients presenting for mitral valve surgery are affected by atrial fibrillation, compared with 1–6% undergoing other forms of cardiac surgery [32]. Moreover, patients undergoing mitral valve surgery have high incidence of postoperative atrial fibrillation (20–60%) compared to other types of cardiac surgery [33,34,35]. These findings could explain the difference in AF proportion between aortic and mitral MPHV patients. Similarly, control rhythm in AF is achieved through antiarrhythmics, including amiodarone [36], which explains why these drugs were more commonly used in mitral MPHVs. Finally, we found a higher proportion of patients treated with warfarin compared to acenocoumarol. This finding could be explained by the higher risk to develop a low anticoagulation quality during treatment with acenocoumarol, as recently shown by a large multicentre cohort of patients with MPHV [37].

NOX2 is expressed in platelets and is involved in platelet activation [38]. In particular, CD40L expression seems directly connected to NOX2 activity through gp91phox activation and ROS production: experimental data showed, in collagen-stimulated platelets of healthy subjects, a significant correlation between O2 levels and CD40L expression, while CD40L expression was suppressed and did not increase after platelet stimulation in subjects affected by X-linked chronic granulomatous disease (X-CGD), an inherited disorder characterized by the absence or deficiency of phagocyte-NADPH oxidase activity due to a mutation of the *CYBB* gene encoding the gp91phox subunit of NOX2 [39]. The relationship between NOX2 and sCD40L was further studied in patients with X-CGD compared with carriers of hereditary deficiency of NOX2, obese women (who express higher levels of NOX2), and healthy subjects, aiming to compare sCD40L levels in different groups with different degrees of NOX2 activation: the results confirmed reduced plasma levels of sCD40L in X-CGD patients and in X-CGD carriers, compared with healthy subjects; moreover, obese women showed higher levels of sCD40L than controls [40]. These findings reinforce the hypothesis of a functional NOX2–CD40L axis in platelets. In particular, the generation of reactive oxygen species by NOX2 appears to be a critical upstream event leading to CD40L surface expression and subsequent release of its soluble form (sCD40L), which exerts pro-thrombotic effects. The impairment of CD40L expression in NOX2-deficient conditions, as observed in X-CGD, underscores the role of NOX2-derived ROS in platelet activation [40]. Furthermore, NOX2-driven CD40L expression could be a key link between oxidative stress and platelet-mediated vascular inflammation. NOX2 promotes the expression of CD40L, amplifying platelet interactions with endothelial cells and leukocytes and enhancing the recruitment and activation of immune cells at sites of vascular injury [41]. Taken together, these data not only highlight the role of NOX2 in modulating platelet activation but also identify this oxidase as a potential therapeutic target to modulate CD40L-mediated inflammatory responses.

### 4.1. Clinical Implications

Our study provided evidence for the first time of enhanced NOX2-mediated OxS in patients with MPHVs. Previous studies have explored how atorvastatin decreases OxS and platelet activation via NOX2 inhibition [42,43]. Moreover, a recent study showed that sodium–glucose co-transporter-2 inhibitors (SGLT2-i) exert an antiplatelet and antithrombotic effect through NOX2 downregulation [44]. These studies did not include MPHV patients, hence their results cannot be generalized to our population. For this reason, the potential role of statins or SGLT2 inhibitors on NOX2 discussed is only hypothesis-generating, and their potential clinical impact should be further investigated. Proving that NOX2 mediates platelet activation in patients carrying MPHVs, our study gives bench evidence to guidelines’ suggestion to add an antiplatelet agent to VKA therapy in patients undergoing thromboembolism despite adequate INR control [7]. Taken together, these findings may open new opportunities for optimizing medical therapy in MPHV carriers, particularly in those who experience recurrent thrombotic episodes despite adequate anticoagulation [45].

### 4.2. Limitations

The small sample size and the observational, single-center design of this study represent important limitations to our results, and its pilot nature prompts further studies on larger cohorts, ideally based on a multicentric and interventional design.

Moreover, the included patients were mainly Caucasian, so our findings are limited with regard to minorities: even though previous studies about NOX2 activity in different ethnicities are lacking, we cannot exclude that genetic polymorphisms may be involved in its regulation, thus limiting the generalizability of our findings.

Furthermore, sNOX2dp, H_2_O_2_ and sCD40L are involved in oxidative stress, but may also be involved in inflammation. In order to limit confounding factors, we excluded patients with obvious sources of inflammation at the moment of blood sampling, such as infectious and autoimmune diseases or cancer, and patients with recent MPHV implantation. However, it cannot be excluded that our results may be influenced by inflammatory states not detected during the medical visit that preceded the sampling: hence, further studies addressing other markers of oxidative stress, such as F2-isoprostanes or malondialdehyde (MDA), are warranted to corroborate these results. Ideally, a complete evaluation of oxidative stress and inflammation may be performed also by pathological examination of valve tissue, especially in patients with valve dysfunction: we could not perform this analysis during our study, but it could be considered for future research.

Finally, we evaluated platelet activation only through plasma sCD40L and did not consider other markers of platelet activation, such as *p*-selectin, involving other platelet activation pathways.

## 5. Conclusions

In conclusion, in patients with MPHVs, oxidative stress was correlated with higher platelet activation. Furthermore, mitral MPHVs are associated with heightened NOX2-mediated oxidative stress and platelet activation compared to aortic MPHVs.

Further studies are needed to confirm these data and to suggest therapeutic implications.

## Figures and Tables

**Table 1:** Clinical characteristics according to MPHV site implantation.

	Total (*n* = 60)	Aortic (*n* = 30)	Mitral (*n* = 30)	*p*
Age [IQR]	57.2 [48.1–64.4]	54.5 [47.0–61.1]	58.4 [51.5–67.0]	0.197
Female sex (%)	32 (53.3)	14 (46.7)	18 (60.0)	0.301
Atrial fibrillation (%)	25 (42.4)	7 (24.1)	18 (60.0)	0.005
Caucasian (%)	54 (90.0)	28 (93.3)	26 (86.7)	0.389
Hypertension (%)	34 (56.7)	18 (60.0)	16 (53.3)	0.602
Diabetes (%)	10 (16.7)	7 (23.3)	3 (10.0)	0.166
Previous stroke/TIA (%)	5 (8.3)	2 (6.7)	3 (10.0)	0.640
PAD (%)	5 (8.3)	1 (3.3)	4 (13.3)	0.161
Previous MI (%)	4 (6.7)	2 (6.7)	2 (6.7)	1.000
Smoke (%)	7 (15.6)	3 (14.3)	4 (16.7)	0.826
Alcohol (%)	14 (33.3)	7 (31.8)	7 (31.8)	0.827
COPD (%)	6 (10.0)	6 (20.0)	0 (0.0)	0.010
Dyslipidaemia (%)	22 (36.7)	11 (36.7)	11 (36.7)	1.000
Thyroid disease (%)	8 (13.6)	1 (3.3)	7 (24.1)	0.020
Liver disease (%)	2 (4.3)	1 (4.3)	1 (4.3)	0.975
Cancer (%)	7 (11.7)	5 (16.7)	2 (6.7)	0.228
Heart failure (%)	10 (16.7)	6 (20.0)	4 (13.3)	0.488
Therapy				
Warfarin (%)	43 (71.7)	18 (60.0)	25 (83.3)	0.045
Acenocoumarol (%)	17 (28.3)	12 (40.0)	5 (16.7)	
LLD (%)	18 (37.5)	8 (33.3)	10 (41.7)	0.551
ACE-I/ARBs (%)	24 (49.0)	8 (33.3)	16 (64.0)	0.032
Beta-blockers (%)	29 (59.2)	16 (66.7)	13 (52.0)	0.296
CCB (%)	6 (12.2)	3 (12.5)	3 (12.5)	0.957
Antiarrhythmics (%)	12 (24.5)	2 (8.3)	10 (40.0)	0.010
Digoxin (%)	4 (8.2)	2 (8.3)	2 (8.3)	0.966
Amiodarone (%)	8 (16.3)	1 (4.2)	7 (28.0)	0.024
Diuretics (%)	23 (46.9)	9 (37.5)	14 (56.0)	0.195
PPI (%)	31 (63.3)	17 (70.8)	14 (56.0)	0.282

ACE-I/ARBs: angiotensin-converting enzyme inhibitors/angiotensin receptor blockers, CCB: calcium channel blockers, COPD: chronic obstructive pulmonary disease, IQR: interquartile range, LLD: lipid-lowering drugs, MI: myocardial infarction, MPHV: mechanical prosthetic heart valve; PAD: peripheral artery disease, PPI: proton pump inhibitors, TIA: transient ischemic attack.

**Table 2:** Plasmatic levels of oxidative stress and platelet activation according to MPHV position.

	Total	Aortic (*n* = 30)	Mitral (*n* = 30)	*p*
sNOX2dp (pg/mL) [IQR]	25.55 [21.33–30.15]	24.27 [17.30–26.41]	28.69 [25.08–33.18]	0.001
H_2_O_2_ (µM) [IQR]	18.73 [13.89–25.74]	16.73 [12.50–20.87]	22.94 [15.79–27.33]	0.013
sCD40L (ng/mL) [IQR]	4.67 [2.67–6.39]	3.65 [2.14–5.54]	5.61 [3.69–6.89]	0.009

H_2_O_2_: hydrogen peroxide; sCD40L: soluble CD40L; sNOX2dp: soluble peptide of NOX2.

**Table 3:** Spearman’s correlation analysis.

		sNOX2dp	H_2_O_2_	sCD40L
Aortic	sNOX2dp	1	0.720 **	0.443 *
	H_2_O_2_	0.720 **	1	0.438 *
	sCD40L	0.443 *	0.438 *	1
Mitral	sNOX2dp	1	0.800 **	0.521 **
	H_2_O_2_	0.800 **	1	0.300
	sCD40L	0.521 **	0.300	1

H_2_O_2_: hydrogen peroxide; sCD40L: soluble CD40L; sNOX2dp: soluble peptide of NOX2. ** *p* < 0.01, * *p* < 0.05.

**Table 4:** Stepwise multivariable linear regression of factors associated with oxidative stress biomarkers.

	Biomarker	B	95% Confidence Interval		*p*-Value
Aortic vs. Mitral	sNOX2dp	−5.042	−8.752	−1.332	0.009
Aortic vs. Mitral	H_2_O_2_	−3.867	−7.082	−0.652	0.019
Aortic vs. Mitral	sCD40L	−1.722	−2.851	−0.592	0.003

Adjusted for atrial fibrilllation, thyroid disease, type of vitamin K antagonist, chronic obstructive pulmonary disease.

## Data Availability

The data presented in this study are available on request from the corresponding author. The data are not publicly available due to privacy restrictions.

## Abbreviations

The following abbreviations are used in this manuscript:

ACE-i/ARBs	angiotensin-converting enzyme inhibitors/angiotensin receptor blockers
AF	atrial fibrillation
CCB	calcium channel blockers
COPD	chronic obstructive pulmonary disease
HF	heart failure
INR	International Normalized Ratio
H_2_O_2_	hydrogen peroxide
IQR	interquartile range
LLP	lipid-lowering drugs
MI	myocardial infarction
MPHVs	mechanical prosthetic heart valves
OxS	oxidative stress
PAD	peripheral artery disease
PPI	proton pump inhibitors
ROS	radical oxygen species
sCD40L	soluble CD40L
SD	standard deviation
SGLT2-i	sodium–glucose co-transporter-2 inhibitors
sNOX2dp	soluble peptide of NOX2
TIA	transient ischemic attack
TTR	time in therapeutic range
VKAs	vitamin K antagonists
